# An Optimized Pentaplex PCR for Detecting DNA Mismatch Repair-Deficient Colorectal Cancers

**DOI:** 10.1371/journal.pone.0009393

**Published:** 2010-02-24

**Authors:** Ajay Goel, Takeshi Nagasaka, Richard Hamelin, C. Richard Boland

**Affiliations:** 1 Division of Gastroenterology, Department of Internal Medicine, Baylor University Medical Center and Charles A. Sammons Cancer Center, Dallas, Texas, United States of America; 2 INSERM, U762, Paris, France; City of Hope National Medical Center, United States of America

## Abstract

**Purpose:**

Microsatellite instability (MSI) is used to screen colorectal cancers (CRC) for Lynch Syndrome, and to predict outcome and response to treatment. The current technique for measuring MSI requires DNA from normal and neoplastic tissues, and fails to identify tumors with specific DNA mismatch repair (MMR) defects. We tested a panel of five quasi-monomorphic mononucleotide repeat markers amplified in a single multiplex PCR reaction (pentaplex PCR) to detect MSI.

**Experimental Design:**

We investigated a cohort of 213 CRC patients, comprised of 114 MMR-deficient and 99 MMR-proficient tumors. Immunohistochemical (IHC) analysis evaluated the expression of MLH1, MSH2, PMS2 and MSH6. MSI status was defined by differences in the quasi-monomorphic variation range (QMVR) from a pool of normal DNA samples, and measuring differences in allele lengths in tumor DNA.

**Results:**

Amplification of 426 normal alleles allowed optimization of the QMVR at each marker, and eliminated the requirement for matched reference DNA to define MSI in each sample. Using ≥2/5 unstable markers as the criteria for MSI resulted in a sensitivity of 95.6% (95% CI = 90.1–98.1%) and a positive predictive value of 100% (95% CI = 96.6%–100%). Detection of MSH6-deficiency was limited using all techniques. Data analysis with a three-marker panel (BAT26, NR21 and NR27) was comparable in sensitivity (97.4%) and positive predictive value (96.5%) to the five marker panel. Both approaches were superior to the standard approach to measuring MSI.

**Conclusions:**

An optimized pentaplex (or triplex) PCR offers a facile, robust, very inexpensive, highly sensitive, and specific assay for the identification of MSI in CRC.

## Introduction

Microsatellite instability (MSI), which is defined as the accumulation of insertion-deletion mutations at short repetitive DNA sequences (or ‘microsatellites’) is a characteristic feature of cancer cells with DNA mismatch repair (MMR) deficiency [1]. Inactivation of any of several MMR genes, including *MLH1*, *MSH2*, *MSH6* and *PMS2*, can result in MSI. Originally, MSI was shown to correlate with germline defects in MMR genes in patients with Lynch syndrome (LS), where >90% of colorectal cancer (CRC) patients exhibit MSI [2], [3]. It was later recognized that MSI also occurs in ∼12% of sporadic CRCs occurring in patients that lack germline MMR mutations, and MSI in these patients is due to promoter methylation-induced silencing of the *MLH1* gene expression [4]. Determination of MSI status in CRC has clinical use for identifying patients with germline defects predisposing to MMR-deficiency. Additionally, MSI status has prognostic and therapeutic implications, because MSI CRCs typically have a better prognosis, and these cancers are less responsive to 5FU-based adjuvant chemotherapy [5].

Since its initial discovery more than a decade ago, the methods and criteria to determine MSI in CRC have constantly evolved. However, there is still a lack of consensus on the use of various MSI assays that are more robust, inexpensive and would result in MSI analyses that best represents MMR-deficiency in laboratories worldwide [6]. In an effort to unify MSI analysis in CRC, in 1997 an National Cancer Institute (NCI) workshop recommended using a reference panel of five MSI markers that consisted of 2 mononucleotide repeat markers (BAT26 and BAT25) and 3 dinucleotide repeat markers (D2S123, D5S346 and D17S250) [7]. In a follow-up NCI workshop, the panel recognized some of the limitations of the original markers, primarily due to the inclusion of the 3 dinucleotide markers [8]. First, it was recognized that the dinucleotide repeat markers were more suitable for identifying MSI-L tumors, while mononucleotide repeat markers were more specific and sensitive for the determination of MSI (or MSI-H) CRCs [9]. Second, due to the polymorphic nature of dinucleotide markers, these required the availability of not just tumor but matching normal DNA from each individual to interpret MSI results. It has been shown that a panel of five quasi-monomorphic mononucleotide repeat markers in a pentaplex PCR obviate the need for normal DNA from each CRC patient, and may offer better specificity and sensitivity than the NCI-panel markers [10].

Unfortunately, in spite of its obvious strengths, the pentaplex MSI approach has gained limited acceptance for MSI-based screening of CRCs. There may be several reasons for this, including a lack of clear understanding on the technical aspects and independent validation of this assay. This study addresses this concern by validating the accuracy of the pentaplex-panel markers in a large series of MMR-proficient and deficient CRCs by analyzing PCR-amplified profiles of each marker in both tumor and matching normal DNA. Herein, we demonstrate a highly sensitive and specific pentaplex PCR assay that requires one-time optimization of quasi-monomorphic variation range (QMVR) for each marker in normal DNA. We provide evidence that an optimized pentaplex PCR assay should be the preferred method for MSI evaluation in clinical and research laboratories, as it is rapid, economical, highly sensitive and specific for detecting MMR-deficient CRCs and obviates the need for reference normal DNA.

## Materials and Methods

### Ethics Statement

All patients provided written informed consent and the study was approved by institutional review boards of Baylor University Medical Center, Dallas, USA; University of Heidelberg, Heidelberg, Germany; and the Okayama University Hospital, Okayama, Japan.

### Tissue Specimens

Tumor and matching germline DNA was collected from 213 patients diagnosed with CRC at three different institutions: 1) Baylor University Medical Center, Dallas, TX, USA 2) University of Heidelberg, Heidelberg, Germany and 3) Okayama University Hospital, Okayama, Japan. Among this cohort, 114 tumors were MMR-deficient, and included 50 CRCs with loss of MLH1, 48 with loss of MSH2 and 8 cases each with the exclusive loss of PMS2 or MSH6 proteins. The remaining 99 cases were MMR-proficient.

### MMR Protein Immunohistochemistry

We examined protein expression for MLH1, MSH2, PMS2, and MSH6 in 213 tumor tissues by immunohistochemical (IHC) staining using DAKO EnVision System-HRP polymer system kit (Dako Cytomation Inc., Carpinteria, CA). Tissue sections were probed with appropriate dilutions of mouse monoclonal antibodies against MLH1 (clone 13271A, BD Pharmingen, San Diego, CA), MSH2 (clone FE11, Oncogene Research Products, Boston, MA), PMS2 (clone A37, BD Pharmingen San Diego, CA), and MSH6 protein (clone 44, BD Transduction Laboratories, Lexington, KY). Tumor cells were scored negative for MMR protein expression only if the epithelial cells within the tumor tissue lacked nuclear staining, while the surrounding stromal cells still showed positive staining.

### Microdissection and DNA Amplification

Serial sections (5 µm) from formalin-fixed paraffin-embedded matched normal and tumor tissues were routinely stained, and representative normal and tumor regions were identified by microscopic examination. Genomic DNA was isolated from the paraffin-embedded tissues using the QIAamp DNA mini kit (Qiagen, Valencia, CA) following separation of tumor and normal tissue by manual microdissection.

### Pentaplex PCR and Quasi-Monomorphic Variation Range (QMVR) Definition

MSI analysis was carried out using five mononucleotide repeat microsatellite targets (BAT-25, BAT-26, NR-21, NR-24 and NR-27) in a pentaplex PCR system [10]. Primer sequences have been described previously, and each sense primer was end-labeled with one of the fluorescent markers: FAM, HEX or NED [11]. Pentaplex PCR was performed in an MJ Research DNA 200 multicycler (Biorad, Hercules, CA). The PCR conditions consisted of an initial 15 min denaturation step at 95°C, followed by 35 cycles at 95°C for 30 s, 55°C for 30 s and 72°C for 30 s, with a final extension at 72°C for 10 min. Amplified PCR products were diluted with formamide, and run on an Applied Biosystems 3100 Avant automated capillary electrophoresis DNA sequencer. Allelic sizes for each of the markers were estimated using GeneMapper 3.1 software (Applied Biosystems, Foster City, CA).

For the determination and validation of the quasi-monomorphic variation range (QMVR) for each of the five MSI markers, PCR amplification profiles were scored individually, and the size of both alleles was determined for each marker and for each tumor individually as described previously [11]. For calculation purposes, due to the monomorphic nature of these markers, we calculated each allele size twice in homozygous samples.

### Determination of Allelic Variations in Tumor DNA Compared to Normal DNA

Next, we investigated whether the availability of matching normal DNA from a CRC patient would enhance the screening performance of the pentaplex PCR in MMR deficient CRCs. We calculated the differences in allelic lengths between tumor and normal DNA for each patient and each MSI marker. In each case, we regarded the shortest allele present in tumor or normal DNA for calculation purposes using the following formula:

*(Difference in allele length)  =  | (Normal DNA allele)–(Tumor DNA allele)| (bp)*

If the PCR fragment from tumor DNA revealed two peaks, we considered the shorter peak representative of tumor DNA.

### MSI Analysis by NCI-Panel Markers

To compare the sensitivity and specificity of pentaplex PCR with the original NCI-panel of markers (BAT25, BAT26, D2S123, D5S346 and D17S250), we performed MSI analyses on a subset of 86 MMR-deficient CRCs and 37 MMR proficient CRCs using both approaches [7]. Primers for each of the 5 markers were previously described [12], [13].

### Statistical Analyses

We used logistic regression analysis to examine the diagnostic performance for MMR deficient CRCs utilizing different strategies to define MSI. To examine the relationship between individual MSI markers, a multivariate correlation and hierarchical clustering analysis was performed using standardized absolute difference length between tumor allele and the germline/normal allele. Analyses were performed using JMP (version 6.0, SAS Institute). All reported P values are two-sided and P<0.05 was considered statistically significant.

## Results

### MMR-Deficient and MMR-Proficient CRCs

To determine the accuracy of a pentaplex PCR system for detecting MMR deficient CRCs, we investigated a cohort of 213 CRCs which comprised of 114 MMR-deficient and 99 MMR-proficient tumors. **Table 1** lists the clinical features of MMR-proficient and –deficient CRCs.

**Table 1:** Characteristics of CRC patients according to MMR protein expression status.

MMR expression status	Mean Age (95%CI)	Male, *n* (%)	Amsterdam Criteria II Positive, *n* (%)
MLH1 Deficient (n = 50)	44.0 (40.0–48.1)	35 (70)	46 (92)
MSH2 Deficient (n = 48)	43.3 (39.9–46.7)	32 (68)	45 (94)
PMS2 Deficient (n = 8)	44.0 (29.0–60.0)	4 (50)	0 (0)
MSH6 Deficient (n = 8)	33.3 (27.5–39.0)	3 (38)	0 (0)
MMR Proficient (n = 99)	47.2 (43.8–50.7)	49 (49)	0 (0)

### Determination and Optimization of QMVR for Each Marker by Normal DNA

**Figure 1:**
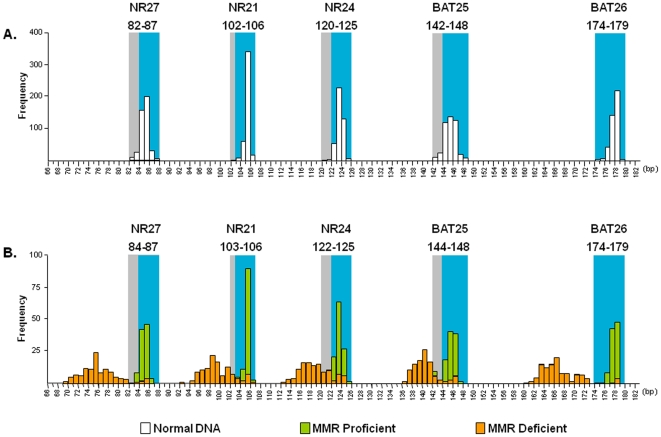
Frequency of allele size distribution for the five pentaplex markers. A) Allele size distribution (in base pairs) from 213 normal DNA specimens. For each marker, blue shading indicates the adjusted QMVR, while the gray shading indicates the entire range of allelic size obtained from 426 germline alleles. B) Distribution of allele sizes in MMR-deficient (orange) and MMR-proficient (green) CRCs.

Although the five mono-repeat markers in pentaplex PCR have been suggested to be highly monomorphic in germline DNA from a wide spectrum of populations worldwide, the success of this MSI assay heavily relies on the accurate determination of QMVR for each marker in normal DNA [11]. Theoretically the QMVR for each marker should be constant in each experimental setting, but data indicates that specific instrumentation or reagents may partly influence allele size measurements for each marker [11]. This mandates one-time careful determination of QMVR in germline DNA prior to tumor MSI analysis. We PCR amplified 426 alleles from 213 normal DNA specimens to determine the QMVR for each MSI marker. As shown in **Figure 1**, the polymorphic range for each MSI marker in normal DNA is as follows: NR 27 (82–87 bp), NR21 (102–106 bp), NR24 (120–125 bp), BAT25 (142–148 bp), and BAT26 (174–179 bp). The most common allele for each of the markers was as follows: NR27 (85 bp), NR21 (105 bp), NR24 (123 bp), BAT25 (145 bp) and BAT26 (178 bp). In contrast to previous studies which used healthy subjects to generate QMVR for each marker [11], QMVR values in our study were based on a large series of matching normal DNA samples obtained from CRC patients. We noticed that our most common alleles were shorter for each marker (BAT26 by 1 bp, NR21 & NR27 by 2 bp and BAT25 & NR24 by 3 bp) and therefore optimized QMVR were shifted slightly leftwards as indicated by grey shaded areas in **Figure 1A**. Thereafter, we considered a tumor to be positive for allelic variation (or unstable) at a given marker when tumor allele sizes did not fall within the optimized QMVR. Supporting the specificity of our newly optimized QMVR, we noted that almost all amplification profiles from all MMR-proficient tumors fell within this range, while almost all MMR-deficient tumors showed large allelic variations at multiple markers and were outside of this range (**Figure 1B**).

### Determination of Allelic Variations in Tumor DNA for Each Marker and Its Relationship to MMR Status in CRC

We noted that due to significant concordance between MMR IHC and MSI results at each marker, we utilize IHC information for the calculation of cut-off thresholds that corresponded with loss of DNA MMR protein expression and MMR-deficiency. We defined cut-offs for each of the five markers by determining specific allele length variations in which at least 95% of tumors were within QMVR (showed no instability) and were simultaneously MMR-proficient according to IHC results. In this manner, we successfully determined that an allelic difference of >3 bp between the tumor and corresponding normal DNA for both BAT25 and BAT26 was diagnostic for a MSI-positive tumor. Similarly, a difference of >2 bp in tumor versus germline DNA for NR21, NR24, and NR27 was considered positive for defining MSI-positivity (gray shaded square box in **Figure S1**).

### Performance Characteristics of *Individual* Pentaplex Markers for the Identification of MMR-Deficient CRCs

We next examined the performance characteristics of individual pentaplex markers, particularly the relatively understudied NR-markers for identifying MMR-deficient CRCs (**Table 2**). Our analyses using ‘QMVR values alone’ clearly highlighted the robustness of various mono-markers to detect MMR-deficient CRCs with a *sensitivity* that varied from 86.8% to 94.7%, and a *specificity* of 96.0% to 100%, for identifying MMR proficient CRCs. Of interest, when results were re-analyzed using ‘data from the matching normal DNA’, the results were strikingly similar, wherein each marker displayed a *sensitivity* range of 85.1% to 95.6% for identifying MMR-deficient CRCs, and a *specificity* of 95.0% to 100% for detecting MMR-proficient CRCs.

**Table 2:** Performance characteristics of each MSI Marker by different strategies for the identification of MMR-Deficient CRCs.

Marker	Reference	Sensitivity % (95%CI)	Specificity % (95%CI)	PPV % (95%CI)#	NPV % (95%CI)+
BAT25	QMVR	90.4 (83.5–94.5)	96.0 (90.1–98.4)	96.2 (90.8–98.5)	89.6 (82.4–94.1)
	Normal DNA	86.0 (78.4–91.2)	97.0 (91.5–99.0)	97.0 (91.6–99.0)	85.7 (78.0–91.0)
BAT26	QMVR	94.7 (89.0–97.6)	97.0 (91.5–99.0)	97.3 (92.4–99.1)	94.1 (87.8–97.3)
	Normal DNA	95.6 (90.1–98.1)	97.0 (91.5–99.0)	97.3 (92.4–99.1)	95.1 (88.9–97.9)
NR21	QMVR	87.7 (80.4–92.5)	100 (96.3–100)	100 (96.3–100)	87.6 (80.3–92.5)
	Normal DNA	89.5 (82.5–93.9)	100 (96.3–100)	100 (96.4–100)	89.2 (82.0–93.7)
NR24	QMVR	86.8 (79.4–91.9)	99.0 (94.5–99.8)	99.0 (94.6–99.8)	86.7 (79.2–91.8)
	Normal DNA	85.1 (77.4–90.5)	95.0 (88.7–97.8)	95.1 (89.0–97.9)	84.7 (76.8–90.2)
NR27	QMVR	93.9 (87.9–97.0)	99.0 (94.5–99.8)	99.1 (94.9–99.8)	93.3 (86.9–96.7)
	Normal DNA	94.7 (98.0–97.6)	96.0 (90.1–98.4)	96.4 (91.2–98.6)	94.1 (87.6–97.2)

# PPV  =  positive predictive value.

+ NPV  =  negative predictive value.

### An Optimized Pentaplex PCR Does Not Require Matching Normal DNA to Detect MMR-Deficient CRC

**Figure 2:**
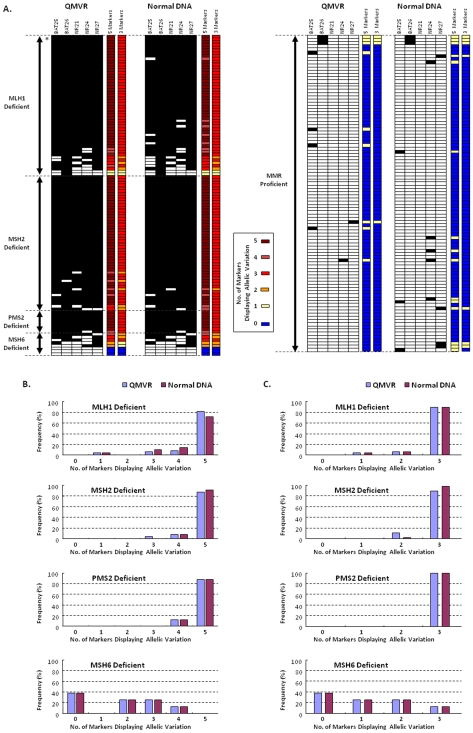
Performance characteristics of pentaplex markers based on QMVR, availability of normal DNA, and numbers of markers required to determine MSI in colorectal cancers. **A**) The figure illustrates the performance of the pentaplex mononucleotide-repeat maker panel when defining the MSI status of tumor DNA by “QMVR only” (when matched normal DNA was not available) and by “Normal DNA” by subtracting the germline allele lengths from tumor for each tumor. Data in the two panels on the left is from MMR-deficient tumors, while the other two panels on the right represent MMR-proficient CRCs. (* indicates one case with loss of both MLH1 and MSH2). Black squares indicate a tumor positive for allelic variation (i.e., unstable) and white squares indicate a tumor negative for any allele variations (i.e., stable). **B**) Shows the frequency of MMR-deficient tumors with number of markers displaying allelic variations when data were analyzed from all ***five*** pentaplex markers. **C**) Shows the frequency of MMR-deficient tumors with number of markers displaying allelic variations when data were analyzed from just ***three*** pentaplex markers (BAT26, NR21 and NR27).

We next analyzed the performance of all mono-markers in the pentaplex PCR assay in both MMR deficient and –proficient CRCs using two approaches: first, using ‘QMVR results alone’ (**Figure 2A** left panels and **Table 3**), and second, when data were available from the ‘matching normal DNA from CRC patients’ (**Figure 2A**, right panels and **Table 4**). When allelic variations at ≥3 of 5 markers was defined as diagnosis of MSI, both strategies displayed 93.9% (CI, 87.9%–97.0%) sensitivity for identification of MMR-deficient CRCs and 100% (CI, 96.3%–100%) specificity for MMR-proficient CRCs. With respect to correlation of MSI data with MMR protein expression status, both strategies demonstrated similar sensitivity for tumors with MLH1 (96.0%), MSH2 (100%), and PMS2 (100%) deficiency. However, neither approach was sufficiently robust to detect MSH6-deficiency and identified only 37.5% (3/8) of MSH6-deficient CRCs (**Figure 2B**). Contrarily, when MSI-H was defined by instability at ≥2 of 5 markers, both strategies demonstrated slightly improved sensitivity for MMR-deficient CRCs (95.6%; CI, 90.1%–98.1%) and same specificity for MMR-proficient CRCs (100%; CI, 96.3%–100%). But this improvement was only due to increased sensitivity for detecting MSH6-deficient tumors (62.5%; 5 of 8 CRCs), without any associated change in sensitivity for identification of MLH1 (96.0%), MSH2 (100%), and PMS2 (100%) deficient (**Figure 2B**). Hence, the optimized pentaplex assay is highly specific and sensitive for detecting MMR-deficient CRCs, and that the availability of normal DNA from a CRC patient does not necessarily enhance its performance. Additionally, using a cut-off for instability at ≥2/5 markers to define MSI results in maximal sensitivity and specificity for this assay, particularly for samples mutant for MSH6.

**Table 3:** Performance characteristics of Pentaplex PCR system with reference to “QMVR” for Identification of MMR-deficient CRCs.

No. of Markers Displaying Allelic Variation	The *Five* Marker Panel*				The *Three* Marker Panel**			
	Sensitivity (%)	Specificity (%)	PPV (%)#	NPV (%)+	Sensitivity (%)	Specificity (%)	PPV (%)#	NPV (%)+
5	78.9 (70.6–85.4)	100 (96.3–100)	100 (95.9–100)	80.5 (72.6–86.5)				
4	87.7 (80.4–92.5)	100 (96.3–100)	100 (96.3–100)	87.6 (80.3–92.5)				
3	93.9 (87.9–97.0)	100 (96.3–100)	100 (96.5–100)	93.4 (87.0–96.8)	85.1 (77.4–90.5)	100 (96.3–100)	100 (96.2–100)	85.3 (77.8–90.6)
2	95.6 (90.1–98.1)	100 (96.3–100)	100 (96.6–100)	95.2 (89.2–97.9)	93.9 (87.9–97.0)	100 (96.3–100)	100 (96.5–100)	93.4 (87.0–96.8)
1	97.4 (92.5–99.1)	90.9 (83.6–95.1)	92.5 (86.4–96.0)	96.8 (90.9–98.9)	97.4 (92.5–99.1)	96.0 (90.1–98.4)	96.5 (91.4–98.6)	96.9 (91.4–99.0)

Results are expressed as percentages (%), with 95% confidence intervals in parentheses.

*The Five Pentaplex Marker Panel composed of BAT25, BAT26, NR21, NR24 and NR27 markers.

**The Three Pentaplex Marker Panel composed of BAT26, NR21 and NR27 markers.

# PPV  =  positive predictive value.

+ NPV  =  negative predictive value.

**Table 4:** Performance characteristics of Pentaplex PCR system with reference to “normal DNA” for the identification of MMR-deficient CRCs.

No. of Markers Displaying Allelic Variation	The *Five* Marker Panel*				The *Three* Marker Panel**			
	Sensitivity (%)	Specificity (%)	PPV (%)#	NPV (%)+	Sensitivity (%)	Specificity (%)	PPV (%)#	NPV (%)+
5	76.3 (67.7–83.2)	100 (96.3–100)	100 (95.8–100)	78.6 (70.6–84.8)				
4	87.7 (80.4–92.5)	100 (96.3–100)	100 (96.3–100)	87.6 (80.3–92.5)				
3	93.9 (87.9–97.0)	100 (96.3–100)	100 (96.5–100)	93.4 (87.0–96.8)	88.6 (87.5–93.2)	100 (96.3–100)	100 (96.3–100)	88.4 (81.1–93.1)
2	95.6 (90.1–98.1)	100 (96.3–100)	100 (96.6–100)	95.2 (89.2–97.9)	93.9 (87.9–97.0)	100 (96.3–100)	100 (96.5–100)	93.4 (87.0–96.8)
1	97.4 (92.5–99.1)	84.9 (76.5–90.6)	88.1 (81.3–92.7)	96.6 (90.3–98.8)	97.4 (92.5–99.1)	92.9 (86.1–96.5)	94.1 (88.3–97.1)	96.8 (91.1–98.9)

Results are expressed as percentages (%), with 95% confidence intervals in parentheses.

*The Five Pentaplex Marker Panel composed of BAT25, BAT26, NR21, NR24 and NR27 markers.

**The Three Pentaplex Marker Panel composed of BAT26, NR21 and NR27 markers.

# PPV  =  positive predictive value.

+ NPV  =  negative predictive value.

### Association between Pentaplex Mononucleotide Repeat Markers

**Figure 3:**
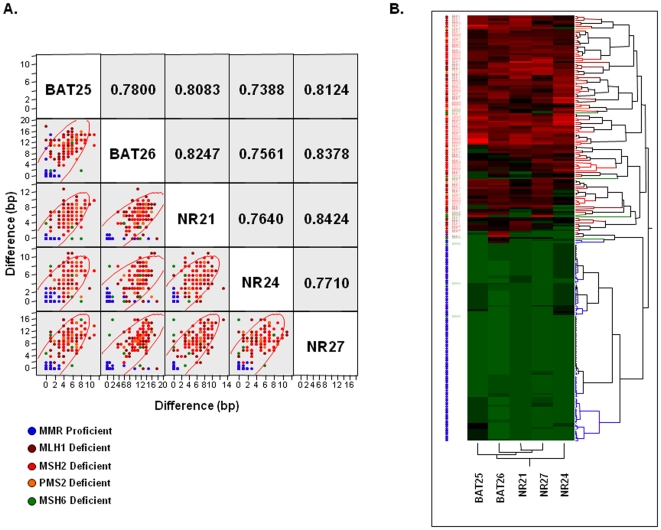
Correlation between various mono-nucleotide markers in the pentaplex PCR. **A**) A scatter-plot matrix demonstrating the pair-wise correlation coefficient (r) between five microsatellite markers in the cohort of MMR-proficient and deficient CRCs. The Y and X-axis denote absolute differences in allele sizes between the tumor DNA and normal DNA. **B**) The figure shows hierarchical clustering analysis derived from 104 MMR-deficient and 99 MMR-proficient CRCs. The data are presented in matrix format in which the rows represent each CRC and the columns indicate the individual mononucleotide markers. The color scale represents the gradient (green to red) of absolute allele length differences between tumor and germline DNA from QMVR standardized data; green (no differences in allele size between tumor and normal DNA) to red (significant differences in allelic lengths between tumor and normal DNA).

We then asked whether an association exists among individual mononucleotide repeat markers, or whether the instability at each marker was an independent event. For this, we performed multivariate correlation as well as hierarchical clustering analysis by comparing the differences in allelic sizes obtained from tumor and normal DNA (**Figures 3A&B**). All correlation coefficients (r) demonstrated values of over 0.75, suggesting a strong mutual association among the markers. However, when analyzing specific pairwise associations, we observed that BAT26, NR21 and NR27 showed higher correlation coefficients between one another (**Figure 3A**). These observations were reconfirmed upon hierarchical clustering analysis, wherein we noticed that NR24 was farthest from the top of the hierarchical tree, followed by BAT25 in comparison to the BAT26, NR21 and NR27 microsatellite repeats which were more tightly correlated (**Figure 3B**).

### Reduced Marker Combination Is Equally Effective as All Five Markers in the Pentaplex Assay

We questioned whether a reduced panel of markers might be equally effective as all five markers in the pentaplex panel. For this, we re-analyzed the screening performance of pentaplex PCR to detect MMR-deficient CRCs based upon all *five*, or a selected panel of *three* (BAT26, NR21 and NR27) markers for MSI classification analysis (**Tables 3** **&** **4**).

When MSI was defined as instability at ≥1 or ≥2 of 3 markers, once again, comparable degrees of sensitivity (93.9%–97.4%) and specificity (92.9%–100%) were obtained. With respect to MMR protein expression status, both strategies displayed similar sensitivity for tumors with MLH1 (96.0%), MSH2 (100%), and PMS2 (100%) deficiency. However, as noted previously with a *five* marker panel, a cut-off threshold of instability at ≥2 of 3 markers resulted in increased sensitivity for the detection of MSH6 deficient CRCs (62.5%; 5 of 8 tumors; **Figure 2C**). Although the sensitivity of an MSI assay using these criteria is marginally lower 93.9% (CI 87.9%–97.0%) compared to using all five markers 95.6% (CI 90.1%–98.1%), the specificity of this assay remained unchanged (100%, with both marker panels).

### Screening Performance of the Pentaplex Assay Is Better than with the NCI-Panel Markers

**Figure 4:**
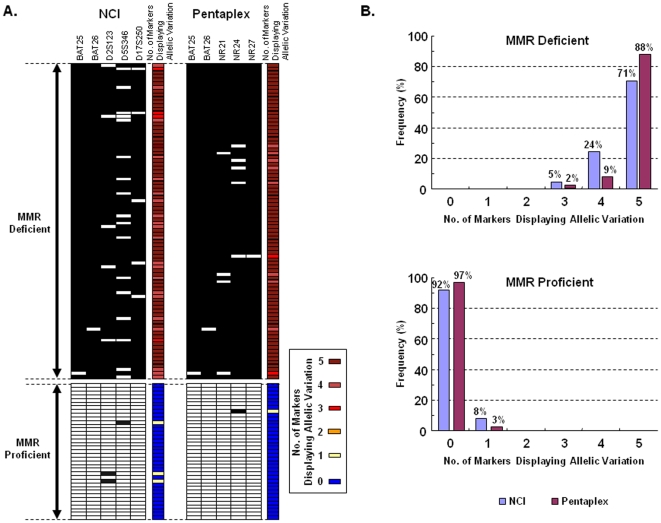
Comparison between pentaplex PCR and NCI panel of markers to determine MMR-deficiency in colorectal cancers. **A**) The figure shows the performance comparison between the NCI panel markers and the QMVR optimized pentaplex PCR. Black squares indicate a tumor positive for allelic variation (or unstable) and white squares indicate a tumor negative for any allele variations (or stable). The dinucleotide repeat markers (D2S123, D5S346 and D17S250) are less robust than the mononucleotide repeats for detecting MSI. **B**) The figure illustrates the frequency of tumors with number of markers displaying allelic variations in MMR-deficient and proficient CRCs. As indicated, pentaplex PCR shows a higher sensitivity and specificity compared to NCI panel of markers, and the distribution of altered markers is unambiguously bimodal.

The NCI-panel of MSI markers (2 mono markers; BAT25 & BAT26 and 3 dinucleotide markers; D3S1023, D5S346 & D17S250) is currently the standard for MSI-determination in CRCs [7]. The dinucleotide markers in this panel require simultaneous amplification of matched normal DNA for the same patient with CRC, and are better suited for detecting MSI-L than MSI tumors. Since pentaplex markers are quasi-monomorphic, we compared the screening performance of these two MSI assays for the identification of the two most commonly defective MMR proteins, MLH1 and MSH2 in our collection of CRCs. As shown in **Figures 4A & B**, the pentaplex markers demonstrated better or comparable sensitivity and specificity to the NCI panel markers for the identification of MMR-deficient CRCs. Given this scenario, the pentaplex PCR offers tremendous overall advantage over NCI-markers, as it is more rapid, utilizes a single PCR reaction, obviates the need for normal DNA, is less expensive and is highly accurate.

## Discussion

The goal of this study was to develop a rapid and highly accurate MSI assay that can be adapted in any laboratory equipped with an automated DNA sequencer. Herein, we optimized and validated the usefulness of five mononucleotide microsatellite markers that can be amplified in a single pentaplex PCR reaction for MSI determination in a large series of MMR-proficient and deficient CRCs.

MSI analysis with the NCI-panel of five microsatellite markers (2 mono- and 3 di-nucleotide repeats) still appears to be a preferred method in most clinical and research laboratories. Unfortunately, although multiple studies have repeatedly shown that mononucleotide MSI markers offer higher accuracy for detecting MSI-H or MMR-deficient tumors [10], this approach has not gained sufficient recognition or acceptance. One of the key technical challenges of this assayis the need for careful one-time optimization of QMVR for each mono-marker. This is because allelic size estimation for these quasi-monomorphic markers can be influenced by the use of specific reagents or the sequencing machine [14]. Supporting this concept, the QMVR for all markers in our patient population differed by a few base pairs than what had been reported previously [11], [14]. We believe that the QMVRs in our study are more robust, as these were obtained from the matching normal/germline DNA from a large series of CRC patients, instead from healthy individuals as reported previously [11], [14]. Previous studies have highlighted the usefulness of BAT25 and BAT26 markers to identify MSI-positive CRCs [9], however, there is limited understanding on the sensitivities and positive predictive values of the NR-markers. A recent report indicated that a screen based on an assessment of only BAT26 and NR24 may be effective for the detection of MMR-deficient CRCs [15]. In fact, BAT26 had the highest sensitivity and positive predictive value in our cohort of MMR-deficient CRCs. On the contrary, pairwise correlation and hierarchical clustering analysis in our study clearly showed weakest predictive values for NR24 (and BAT25), compared to the remaining *three* markers (BAT26, NR21, and NR27). BAT26 is a quasi-monomorphic marker which is located immediately 3’ to *MSH2* exon 5, and is considered to be very sensitive and specific for MSI testing. However, large deletions in *MSH2* that include the BAT26 locus are not uncommon in CRC, and in such instances, although the tumor is MMR-deficient, PCR at the BAT26 locus will result in the false negative amplification of the wild-type alleles from the normal cells in the tumor mass [16]. These data caution against the conventional wisdom that although BAT26 is frequently used for MSI-determination, using BAT26 alone, or in conjunction with a less accurate marker such as NR24 can underestimate MSI, and will preclude detection of potential MMR-deficient CRCs. In addition, our observation of the high sensitivity and positive predictive value for a reduced panel of *three* markers (BAT26, NR21 and NR27) versus all *five* makers have economic implications for future MSI-based assays.

Another critical issue with the use of the pentaplex assay is lack of agreement on the minimum number of unstable markers required to classify a tumor as MSI. In this context, the original report suggested ≥3/5 unstable markers in tumor DNA would define a MSI-positive CRC [10], while a subsequent study suggested that instability at only ≥2/5 markers was sufficient to detect a MMR-deficient CRC [15]. We revisited this issue by analyzing data through multiple approaches and our results demonstrate that the sensitivity and specificity of the pentaplex PCR was unaffected regardless of whether we considered a cut-off of ≥2 or ≥3 of 5 markers for the detection of MLH1, MSH2 and PMS2-deficient CRCs. However, we propose that a criterion of ≥2 of 5 unstable markers is more accurate, as it enhanced the screening performance of the assay by identifying additional MSH6-deficient tumors without adding any false positives.

One of the limitations of the NCI-panel of markers is its inability to identify MSH6-deficient CRCs. Our data indicate that the use of mono-markers in the pentaplex panel can identify the majority of MSH6 deficient CRCs. This is of significance because the MutSα complex, a heterodimer of MSH2 and MSH6, preferentially recognizes base/base mismatches as well as small insertion/deletion loops containing 1 or 2 unpaired nucleotides in the DNA sequence and directs the repair of these lesions [17]. Therefore, one would expect that the functional loss of MutSα due to MSH6-deficiency would lead to preferential instability in the loci containing mononucleotide repeats [18].

In conclusion, we present evidence that favor the use of an optimized pentaplex PCR system to screen for MMR-deficient CRCs. Our data indicate that a one-time optimized QMVR obviates the need for amplification of matched normal DNA to determine instability in the tumor tissue, and that instability at ≥2 of 5 markers provides the most robust strategy to identify MMR-deficient CRCs. Our data suggest that a marker panel consisting of BAT26, NR21 and NR27 markers was as accurate as the five- marker panel for MSI analysis. Importantly, the pentaplex markers showed a higher sensitivity for diagnosing MSH6-deficient CRCs. We propose that this assay will replace existing methodologies and help improve MSI-based CRC screening in the future.

## Supporting Information

Figure S1 Frequency of allele size differences (in bp) between normal and tumor DNA at each marker, and the MSI status determination by QMVR (horizontal bars in blue and red on the left side) as well as by the status of MMR protein expression by IHC (horizontal bars in green and orange on the right side). The numbers on the Y-axis represent the allele sizes difference (in bp) between normal and tumor DNA. The numbers in red reflect the microsatellite instability cut-off ranges determined for each of the markers based upon their deviation from the QMVR range and IHC data.
(1.59 MB TIF)

## References

[1] Bronner CE, Baker SM, Morrison PT, Warren G, Smith LG (1994). Mutation in the DNA mismatch repair gene homologue hMLH1 is associated with hereditary non-polyposis colon cancer.. Nature.

[2] Ionov Y, Peinado MA, Malkhosyan S, Shibata D, Perucho M (1993). Ubiquitous somatic mutations in simple repeated sequences reveal a new mechanism for colonic carcinogenesis.. Nature.

[3] Thibodeau SN, Bren G, Schaid D (1993). Microsatellite instability in cancer of the proximal colon.. Science.

[4] Kane MF, Loda M, Gaida GM, Lipman J, Mishra R (1997). Methylation of the hMLH1 promoter correlates with lack of expression of hMLH1 in sporadic colon tumors and mismatch repair-defective human tumor cell lines.. Cancer Res.

[5] Ribic CM, Sargent DJ, Moore MJ, Thibodeau SN, French AJ (2003). Tumor microsatellite-instability status as a predictor of benefit from fluorouracil-based adjuvant chemotherapy for colon cancer.. N Engl J Med.

[6] Laghi L, Bianchi P, Malesci A (2008). Differences and evolution of the methods for the assessment of microsatellite instability.. Oncogene.

[7] Boland CR, Thibodeau SN, Hamilton SR, Sidransky D, Eshleman JR (1998). A National Cancer Institute Workshop on Microsatellite Instability for cancer detection and familial predisposition: development of international criteria for the determination of microsatellite instability in colorectal cancer.. Cancer Res.

[8] Umar A, Boland CR, Terdiman JP, Syngal S, de la CA (2004). Revised Bethesda Guidelines for hereditary nonpolyposis colorectal cancer (Lynch syndrome) and microsatellite instability.. J Natl Cancer Inst.

[9] Perucho M Correspondence re: C.R. Boland et al., A National Cancer Institute workshop on microsatellite instability for cancer detection and familial predisposition: development of international criteria for the determination of microsatellite instability in colorectal cancer..

[10] Suraweera N, Duval A, Reperant M, Vaury C, Furlan D (2002). Evaluation of tumor microsatellite instability using five quasimonomorphic mononucleotide repeats and pentaplex PCR.. Gastroenterology.

[11] Buhard O, Cattaneo F, Wong YF, Yim SF, Friedman E (2006). Multipopulation analysis of polymorphisms in five mononucleotide repeats used to determine the microsatellite instability status of human tumors.. J Clin Oncol.

[12] Goel A, Arnold CN, Niedzwiecki D, Chang DK, Ricciardiello L (2003). Characterization of sporadic colon cancer by patterns of genomic instability.. Cancer Res.

[13] Goel A, Nagasaka T, Arnold CN, Inoue T, Hamilton C (2007). The CpG island methylator phenotype and chromosomal instability are inversely correlated in sporadic colorectal cancer.. Gastroenterology.

[14] Buhard O, Suraweera N, Lectard A, Duval A, Hamelin R (2004). Quasimonomorphic mononucleotide repeats for high-level microsatellite instability analysis.. Dis Markers.

[15] Xicola RM, Llor X, Pons E, Castells A, Alenda C (2007). Performance of different microsatellite marker panels for detection of mismatch repair-deficient colorectal tumors.. J Natl Cancer Inst.

[16] Pastrello C, Baglioni S, Tibiletti MG, Papi L, Fornasarig M (2006). Stability of BAT26 in tumours of hereditary nonpolyposis colorectal cancer patients with MSH2 intragenic deletion.. Eur J Hum Genet.

[17] Modrich P (2006). Mechanisms in eukaryotic mismatch repair.. J Biol Chem.

[18] Verma L, Kane MF, Brassett C, Schmeits J, Evans DG (1999). Mononucleotide microsatellite instability and germline MSH6 mutation analysis in early onset colorectal cancer.. J Med Genet.

