# Correction: An Optimized Pentaplex PCR for Detecting DNA Mismatch Repair-Deficient Colorectal Cancers

**DOI:** 10.1371/annotation/572bb6d3-0315-40b1-a6d7-ce818809b5ea

**Published:** 2010-03-15

**Authors:** Ajay Goel, Takeshi Nagasaka, Richard Hamelin, C. Richard Boland

The affiliations of the second author, Richard Hamelin, are incorrect. First, affiliation number 2 is incorrect. It should read: INSERM, UMRS 938, Paris, France. Second, an affiliation for this author was omitted. Richard Hamelin is also affiliated with Université Pierre et Marie Curie-Paris 6, Paris, France.

