# Supplementary figures and images for: An Optimized Pentaplex PCR for Detecting DNA Mismatch Repair-Deficient Colorectal Cancers
Source: PLoS One. 2010 Feb 24;5(2):e9393. doi: 10.1371/journal.pone.0009393 (PMC2827558; doi:10.1371/journal.pone.0009393)

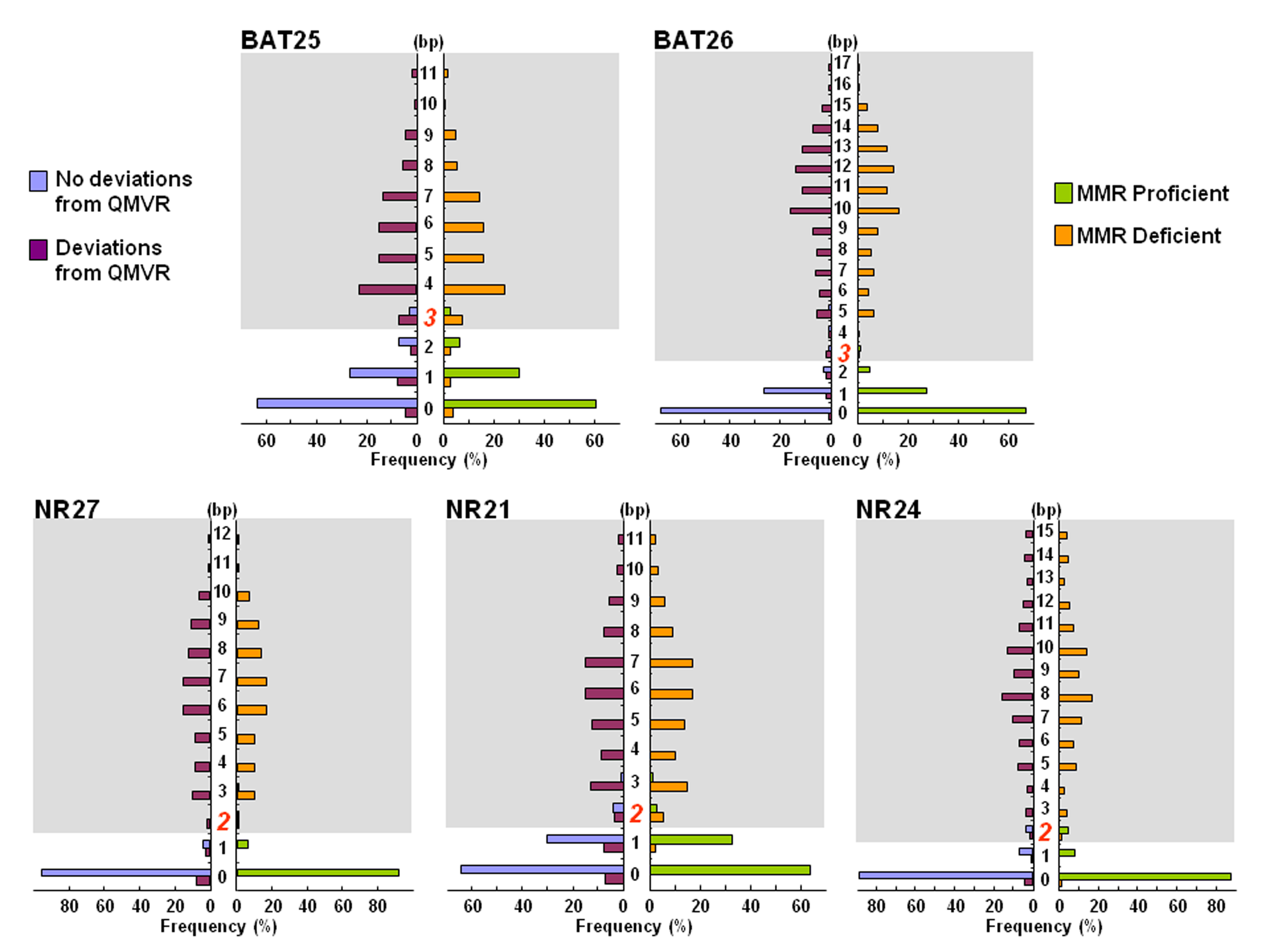

Supplement: Figure S1 — Frequency of allele size differences (in bp) between normal and tumor DNA at each marker, and the MSI status determination by QMVR (horizontal bars in blue and red on the left side) as well as by the status of MMR protein expression by IHC (horizontal bars in green and orange on the right side). The numbers on the Y-axis represent the allele sizes difference (in bp) between normal and tumor DNA. The numbers in red reflect the microsatellite instability cut-off ranges determined for each of the markers based upon their deviation from the QMVR range and IHC data. (1.59 MB TIF) [file pone.0009393.s001.tif]
